# A Case Report of Loperamide-Induced Respiratory Depression in Severe Gastrointestinal Inflammation Secondary to Chemotherapy

**DOI:** 10.7759/cureus.104460

**Published:** 2026-02-28

**Authors:** Joanne Yuen Heng Thong, Rajkumar Satyavolu

**Affiliations:** 1 Department of Critical Care Medicine, Goulburn Valley Health, Shepparton, AUS

**Keywords:** chemo colitis, chemotherapy-related toxicity, gastroduodenitis, hypercapnic respiratory failure, loperamide, oesophagitis, p-glycoprotein

## Abstract

Under normal circumstances, loperamide has minimal systemic absorption due to extensive first-pass metabolism and limited permeability across the intestinal mucosa. However, in the presence of severe gastrointestinal inflammation, compromised mucosal integrity may enhance loperamide’s central opioid effects via altered pharmacokinetics. Although respiratory depression due to therapeutic loperamide use remains sparse, this case accentuates the risks in vulnerable patients. This case report describes a rare case of loperamide-induced respiratory depression in a patient with chemotherapy-induced gastrointestinal mucosal inflammation, and highlights the potential increased systemic absorption and opioid-like toxicity in vulnerable patients receiving high-dose loperamide therapy. A 75-year-old female who was undergoing active chemotherapy developed significant diarrhoea. Endoscopy revealed active inflammation from the oesophagus to the large bowel. She was treated with high-dose loperamide and octreotide. Subsequently, she developed respiratory depression and altered consciousness, which resolved rapidly with naloxone infusion without lasting effects. In such settings, clinicians should exercise caution with high-dose loperamide as compromised mucosal barriers can facilitate systemic drug accumulation and toxicity.

## Introduction

Loperamide is a widely accessible, over-the-counter medication used to treat various forms of diarrhoea, including traveller’s diarrhoea and chronic diarrhoea secondary to irritable bowel syndrome, or to reduce ileostomy output [1]. It is also commonly used as a standard first-line therapy in chemotherapy-induced diarrhoea (CID) [2]. CID is defined as an increase of more than four stools daily in comparison to baseline, with grades of 1 to 5 detailing its severity, ranging from impaired activities of daily living to nutritional insufficiencies and haemodynamic compromise. It is a well-known side effect of chemotherapy, especially in regimens that incorporate fluorouracil (5-FU) and irinotecan [3]. At recommended therapeutic doses up to 16 mg daily, loperamide exerts its effect on peripheral μ-opioid receptors located in the intestinal walls and does not cross the blood-brain barrier. However, at supratherapeutic doses, it overwhelms P-glycoprotein, allowing loperamide to cross the blood-brain barrier, leading to central opioid effects like euphoria, miosis, and respiratory depression [1]. Although it is still rare, there has been an increasing number of case reports depicting cardiotoxicity as a complication of loperamide abuse. It may lead to broadening of QRS complexes, prolonged QTc, ventricular tachycardias (VTs), torsades de pointes, and even sudden cardiac arrest [4,5].

Our case report describes the toxicity effects of loperamide on the central nervous system (CNS) in a patient with CID. Fortunately, the patient did not develop dangerous arrhythmias and the adverse effects were promptly reversed by naloxone. Respiratory depression caused by therapeutic doses of loperamide remains rare but can occur in patients with a severely inflamed gastrointestinal tract, as described here. Our case hopes to highlight the early recognition of such clinical deterioration from an easily reversible cause.

## Case presentation

A 75-year-old female with a history of stage III caecal adenocarcinoma, currently undergoing adjuvant Capox chemotherapy post right hemicolectomy, was admitted to a regional hospital in Victoria for high output diarrhoea with haemodynamic instability and electrolyte derangement. Her other past medical history included hypertension and gastro-oesophageal reflux disease (GORD).

On presentation, she was transferred from a private hospital 14 days after her second cycle of chemotherapy. She presented with nausea, vomiting, diarrhoea, and fever without abdominal pain, with CT findings of stomach and oesophageal dilatation. There was also a 25-cm segment of wall thickening and luminal narrowing of the gastric pylorus - evidence of gastric outlet obstruction. She was commenced on supportive therapy with bowel rest, parenteral nutrition, fluids, proton pump inhibitor, broad-spectrum antibiotics, and electrolyte correction.

**Figure 1:**
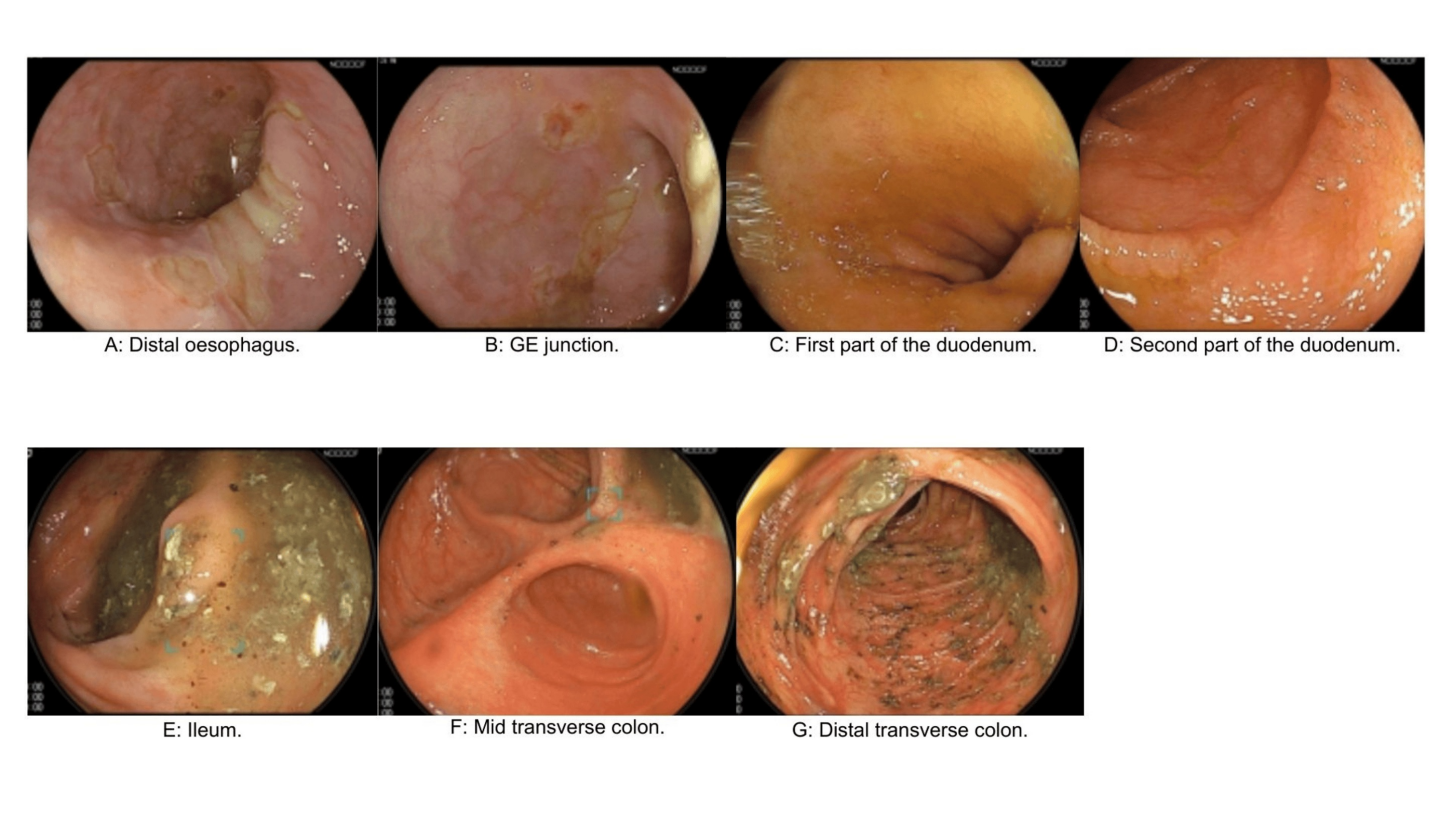
(A-G) Endoscopic images of the distal oesophagus, gastroesophageal (GE) junction, duodenum, ileum, and transverse colon.

On the second day, the patient underwent a gastroscopy and colonoscopy with results showing numerous small ulcers throughout the oesophagus, a large hiatus hernia and grade C reflux oesophagitis, antral gastritis and duodenitis, as well as chronic active colitis, depicted by endoscopic images (Figure 1) and biopsy results below (Table 1). An early stool sample had ruled out bacterial or viral causes of diarrhoea. The impression was chemotherapy-induced gastroduodenitis, enteritis and colitis, causing high volume output. She was commenced on standard doses of loperamide, codeine phosphate, and octreotide to treat the high output diarrhoea; however, she required escalating doses of all three anti-diarrheals for symptom control.

**Table 1:** Biopsy results of endoscopy.

Biopsy location	Findings
Oesophageal ulcer	Mild oesophagitis and a separate fragment of ulcer slough.
Stomach	Mild to moderate chronic active gastritis. *H. pylori* negative.
Duodenum	Moderate active duodenitis.
Neo-terminal ileum	Moderate active ileitis.
Random colon	Focal chronic active colitis.

Unfortunately, her lengthy hospitalisation was compromised by two ICU admissions. The first admission was for fluid-responsive hypovolaemia and aggressive electrolyte replacement with potassium. Intravenous sodium bicarbonate was given for compensated metabolic acidosis. Upon her discharge from the ICU back to the ward, the dosages of anti-diarrheals included 16 mg of oral loperamide four times a day (QID) (64 mg daily), 60 mg of oral codeine phosphate three times a day (TDS) (180 mg daily), and 500 mcg of IV octreotide TDS. Her second ICU admission was triggered by an altered conscious state in the ward, where the patient reported increasing drowsiness. Intravenous naloxone was given to good effect as it was presumed codeine phosphate was causing the drowsiness and was immediately ceased, with the continuation of high-dose loperamide at 64 mg daily and octreotide.

Throughout her stay, she remained significantly drowsy, albeit arousable. However, her general conscious state subsequently declined to be only minimally responsive to noxious stimuli, with a respiratory rate of 8 and acute respiratory acidosis shown on her venous blood gas (Table 2). Naloxone infusion was immediately commenced and ceased within 24 hours, resulting in rapid improvement in the patient’s level of consciousness and resolution of hypercapnia. High-dose loperamide was reduced to 8 mg QID with gradual improvements in her high-output diarrhoea.

**Table 2:** Venous blood gas results before and after naloxone infusion. pCO2: partial pressure of carbon dioxide; pO2: partial pressure of oxygen.

Parameter	pH	pCO2	pO2	Bicarbonate	Lactate
Before naloxone infusion	7.27	71 mmHg	37 mmHg	32 mmol/L	0.4 mmol/L
After naloxone infusion	7.39	49 mmHg	37 mmHg	30 mmol/L	0.9 mmol/L

We attempted to obtain serum loperamide concentrations; however, this investigation is not available at our facility. The patient was later discharged to the ward to continue managing her nutritional intake.

## Discussion

Loperamide is a lipophilic synthetic opioid that functions as a μ-opioid receptor agonist, targeting receptors found on the circular and longitudinal muscles of the intestine. These receptors are abundantly present in the myenteric and submucosal plexus. By binding to them, loperamide inhibits the release of neurotransmitters and interferes with both excitatory and inhibitory signals in motor and secretory pathways, at both pre- and post-synaptic levels. This action suppresses the release of acetylcholine and prostaglandins, leading to a decrease in colonic transit time and reduced peristalsis, allowing for increased contact time for fluid and electrolyte absorption [1].

Loperamide is absorbed from the gastrointestinal system and undergoes first-pass metabolism in the liver. This drug is prevented by the efflux mechanism of P-glycoprotein from crossing the blood-brain barrier; hence, it was released as a widely available, nonprescription drug with minimal opioid toxicity. However, recent studies have come to light showing loperamide’s potential abuse, being used to self-manage opioid withdrawal symptoms and is an inexpensive method to induce euphoria [5]. This has brought to light a syndrome termed loperamide-induced cardiotoxicity, where patients present with different forms of life-threatening arrhythmias when loperamide is consumed in supratherapeutic doses. The proarrhythmic effects of loperamide likely stem from the inhibition of sodium/potassium (Na+/K+) channels in the cardiomyocytes, resulting in delays in depolarisation and QRS prolongation; whereas its metabolite, N-desmethyl-loperamide, inhibits hERG voltage-gated potassium channels, which leads to delays in repolarisation, causing QTc prolongation [1,4,5].

In CID, loperamide remains the standard first-line treatment for grade 1 and 2 CID, with the maximum daily dosage of 24 mg; whereas patients who require hospitalisation for CID should be started on octreotide injections, with increments up to 500 mcg TDS [2,3].

P-glycoprotein plays an important role in the gastrointestinal system as a detoxification mechanism by constantly exporting xenobiotics out of cells. Its expression and function are also dependent on the surrounding gastrointestinal environment, easily influenced by oxidative or inflammatory stress, gut microbiota, and so forth. Studies have shown that the expression of P-glycoprotein is downregulated in acute intestinal inflammation, such as active inflammatory bowel diseases [6]. Hence, one can theorise that, in an actively inflamed state, the gut cannot function fully to absorb loperamide in addition to its already reduced expression of P-glycoprotein, leading to drug concentration accumulation in the systemic circulation.

In our case study, these factors, in addition to a prolonged, over-the-limit dosage, all contributed to the patient’s CNS depression and subsequently a decreased respiratory drive.

Treatment of loperamide toxicity is largely supportive and based on the severity of toxicity. Respiratory depression should be treated with naloxone, with a continuous infusion titrated to maintain adequate respiratory drive. It is also not unreasonable to give activated charcoal in an acute overdose. Cardiac arrhythmias should be managed according to advanced cardiac life support protocols, anti-arrhythmics, and the optimisation of electrolytes such as magnesium and potassium to prevent further arrhythmias. Sodium bicarbonate can be administered as well to reduce sodium channel blockade. One theory even suggested intralipid emulsion therapy to absorb lipophilic loperamide and reduce myocardial binding [5].

## Conclusions

In summary, loperamide toxicity should remain a high index of clinical suspicion, especially in patients presenting with features of opioid intoxication and cardiac dysfunction, as its serum concentrations are not usually available in routine drug screens. Prompt treatment is crucial to prevent worsening of loperamide toxicity. Care should also be taken when prescribing opioids, including weak opioids, in patients with altered gut metabolism, as it may easily lead to drug accumulation and overdose.
